# Colonic Stenting as a Bridge to Surgery Versus Emergency Resection in Left-Sided Malignant Large Bowel Obstruction: A Systematic Review

**DOI:** 10.7759/cureus.110445

**Published:** 2026-06-08

**Authors:** Abdulaziz Alsamani Abdullah Omar, Manahil Awan, Priyanka Kissoonsingh, Shashwat Shetty, Ayan Ali

**Affiliations:** 1 General Surgery, Prince Othman Digna Teaching Hospital, Port Sudan, SDN; 2 Trauma and Orthopedic, University Hospital Birmingham NHS Trust, Birmingham, GBR; 3 Urology, Sandwell and West Birmingham Trust Hospital, Birmingham, GBR; 4 Orthopedics, Hillingdon Hospital, Uxbridge, GBR; 5 Orthopedic Surgery, Dow University of Health Sciences, Civil Hospital Karachi, Karachi, PAK

**Keywords:** bridge to surgery, colonic stent, large bowel obstruction, left-sided colorectal cancer, outcomes, self-expanding metallic stent

## Abstract

Acute left-sided malignant large bowel obstruction represents a high-risk surgical emergency, frequently associated with significant morbidity, the need for stoma formation, and increased perioperative complications. The use of self-expanding metallic stents (SEMS) as a bridge to surgery has gained prominence as it allows decompression of the obstructed bowel, patient stabilisation, and conversion to a planned elective procedure. This systematic review included seven studies (four randomised controlled trials and three observational cohorts; total n = 819) comparing SEMS as a bridge to surgery with emergency resection. Across the evidence included, SEMS was consistently associated with lower rates of stoma formation and improved facilitation of elective surgery, while short-term morbidity and mortality remained comparable between the two approaches. Stent-related complications, including perforation, were relatively uncommon and largely dependent on operator expertise. Long-term oncological outcomes, including overall survival and disease-free survival, were similar between SEMS and emergency surgery groups, suggesting that the use of SEMS does not adversely affect cancer-related outcomes. Functional benefits, such as effective bowel decompression and improved perioperative optimisation, were more evident in the SEMS group, particularly among elderly patients and those with significant comorbidities. Despite variability in study design and outcome reporting, the overall body of evidence supports SEMS as a safe and effective bridge-to-surgery strategy. It offers meaningful short-term clinical advantages without compromising long-term oncological safety in appropriately selected patients with left-sided malignant large bowel obstruction.

## Introduction and background

Colorectal cancer is a leading cause of global cancer-related morbidity and mortality [1]. Acute large bowel obstruction (LBO) develops in approximately 10-20% of affected patients, with the left colon being the most commonly involved site [2]. Obstruction in this region represents a surgical emergency and is associated with significant clinical risk, including colonic distension, potential perforation, and metabolic imbalance, all of which contribute to increased morbidity and mortality. Historically, emergency surgical resection has been the conventional management strategy; however, it is often linked to higher rates of postoperative complications, increased likelihood of stoma formation, and extended hospital stays, particularly in older individuals or those with significant comorbidities [3]. In recent years, self-expanding metallic stents (SEMS) have emerged as an alternative approach, used as a bridge to surgery (BTS) [4]. By relieving the obstruction, SEMS enables physiological stabilisation and allows time for optimisation prior to a planned elective procedure. This staged approach may reduce perioperative risks, lower stoma rates, and improve short-term outcomes. Nevertheless, SEMS placement is not without complications, including perforation, migration, and technical failure, which may impact its safety and effectiveness.

A growing body of evidence, including randomised controlled trials and observational studies, has compared SEMS as a bridge to surgery with immediate emergency resection in patients with left-sided malignant LBO. While some studies report favourable short-term outcomes with SEMS, particularly in reducing stoma formation and postoperative morbidity, the evidence regarding long-term oncological outcomes, such as overall survival and recurrence, remains inconsistent. This study aims to systematically evaluate and compare the clinical and surgical outcomes of colonic stenting as a bridge to surgery versus emergency resection in patients with acute left-sided malignant large bowel obstruction. Primary outcomes include mortality, morbidity, stoma formation, and perioperative complications. Secondary objectives include the assessment of long-term oncological outcomes, such as overall survival and recurrence rates, as well as the identification of gaps in the current evidence to guide future research and clinical practice.

## Review

Materials and methods

Search Strategy

A comprehensive literature search was performed using PubMed, Embase, Scopus, and the Cochrane Library to identify relevant studies published up to 05-March-2026. The search strategy incorporated a combination of keywords and controlled vocabulary (MeSH terms), including “colonic stent,” “bridge to surgery,” “large bowel obstruction,” “left-sided colorectal cancer,” “self-expanding metallic stent,” and “emergency surgery.” Boolean operators (AND/OR) were used to optimise the sensitivity and specificity of the search. In addition, the reference lists of all included articles were manually reviewed to capture any further eligible studies. The search and selection process was conducted in accordance with PRISMA 2020 guidelines to ensure methodological transparency and reproducibility [5].

Eligibility Criteria

Study selection was guided by the PICOS framework [6]. The population comprised adult patients (≥18 years) presenting with acute malignant obstruction of the left colon. The intervention of interest was the use of self-expanding metallic stents (SEMS) as a bridge to surgery, while the comparator was emergency surgical resection, including procedures such as primary anastomosis or Hartmann’s operation. Outcomes of interest included both short-term clinical endpoints, such as mortality, morbidity, stoma formation, and functional recovery, and long-term oncological outcomes, including overall survival and tumour recurrence. Eligible study designs included randomised controlled trials and observational cohort studies. Studies were included if they involved adult patients with left-sided malignant large bowel obstruction and directly compared SEMS as a bridge to surgery with emergency resection. Only articles published in English were considered. Exclusion criteria encompassed case reports, animal studies, editorials, conference abstracts, and previously published systematic reviews or meta-analyses.

Study Selection

All retrieved records were independently screened by two reviewers at the title and abstract level to determine potential eligibility. Articles considered relevant underwent full-text assessment against the predefined inclusion criteria. Any disagreements between reviewers were resolved through discussion, with involvement of a third reviewer where necessary to achieve consensus. The study selection process is illustrated in a PRISMA 2020 flow diagram, which outlines the number of records identified, screened, excluded, and ultimately included in the review.

Data Extraction

Data extraction was conducted independently by two reviewers using a structured and standardised data collection form. Extracted information included study characteristics (author, year, design, and sample size); patient demographics (age, sex, and comorbidities); intervention details (type and timing of SEMS placement); comparator interventions (type of emergency surgery); and outcomes. Clinical outcomes comprised mortality, morbidity, stoma formation, and functional recovery, while oncological outcomes included overall survival, disease-free survival, and recurrence rates. Any discrepancies in extracted data were resolved through discussion to ensure completeness and accuracy.

Risk of Bias Assessment

The methodological quality of the included studies was evaluated using established risk-of-bias tools. Randomised controlled trials were assessed using the Cochrane Risk of Bias 2 (RoB 2) tool [7], which examines domains such as the randomisation process, adherence to intended interventions, completeness of outcome data, outcome measurement, and selective reporting. Observational studies were appraised using the ROBINS-I tool [8], focusing on confounding, selection bias, intervention classification, deviations from intended interventions, missing data, outcome assessment, and reporting bias. An overall risk-of-bias judgement was assigned to each study, and these assessments were considered during interpretation of the findings.

Data Synthesis

Due to heterogeneity across study designs, patient populations, stent techniques, and outcome definitions, a qualitative synthesis was undertaken. Outcomes were grouped into short-term clinical endpoints, such as procedural success, mortality, morbidity, stoma formation, and functional recovery, and long-term oncological outcomes, including overall survival, disease-free survival, and recurrence. Although key numerical findings were summarised in tables, meta-analysis was not performed because of variability in study methodologies and reporting. Patterns and trends across studies were analysed to evaluate the relative safety and effectiveness of SEMS as a bridge to surgery compared with emergency resection.

Registration 

This systematic review followed the PRISMA 2020 reporting guidelines. The protocol was not registered in PROSPERO due to time constraints at the study initiation stage.

Results

Study Selection Process

**Figure 1:**
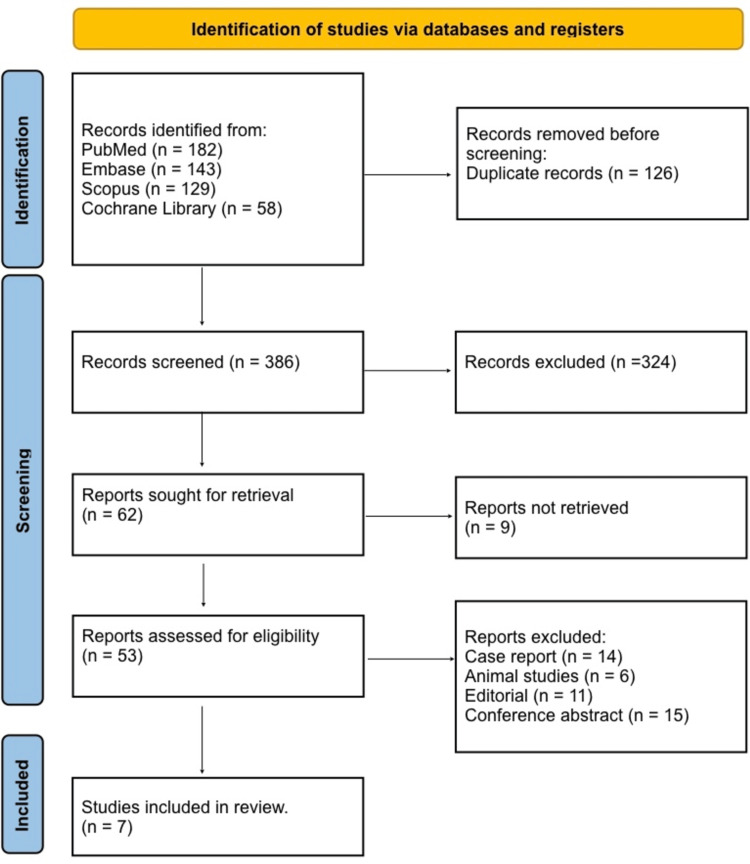
PRISMA 2020 flow diagram

Figure 1 shows that the initial database search yielded a total of 512 records, comprising 182 from PubMed, 143 from Embase, 129 from Scopus, and 58 from the Cochrane Library. After removing 126 duplicates, 386 unique records remained for title and abstract screening, of which 324 were excluded for not meeting the inclusion criteria. Sixty-two articles were selected for full-text review; however, nine could not be accessed. Of the 53 full-text articles assessed, 46 were excluded due to study type, including case reports (n = 14), animal studies (n = 6), editorials (n = 11), and conference abstracts (n = 15). Ultimately, seven studies fulfilled the eligibility criteria and were included in the final systematic review.

Characteristics of the Selected Studies

Table 1 summarises seven studies included in this systematic review, comprising four randomised controlled trials (RCTs) and three observational cohort studies. van Hooft et al. (2011) conducted a multicentre RCT of 98 patients with acute left-sided malignant large bowel obstruction (LBO), comparing SEMS placement as a bridge to elective surgery versus emergency resection; the stent group had higher perforation rates, with no difference in mortality [9]. Wang Y et al. (2019) evaluated 78 patients in a retrospective Cohort and reported no clear mortality or morbidity benefit for SEMS, with early trial termination due to slow recruitment [10]. Ho KS et al. (2011) performed a smaller RCT of 28 patients, demonstrating reduced stoma formation and effective bowel decompression with SEMS, while overall morbidity remained similar to emergency surgery [11]. Among observational studies, Sloothaak et al. (2014) reported comparable long-term overall survival (OS) and disease-free survival (DFS) between SEMS and emergency resection in 73 patients [12]. Arezzo et al. (2017) included 144 patients in an RCT, showing reduced stoma formation and improved quality of life with SEMS, without compromising oncological outcomes [13]. Katsuki R et al. (2020) analysed 212 patients, finding lower stoma rates and similar short-term mortality with SEMS [14]. Finally, Siddiqui A et al. (2017) confirmed in 168 patients that SEMS did not adversely affect long-term survival or recurrence [15].

**Table 1:** Characteristics of included studies comparing colonic stenting versus emergency resection SEMS: Self-expanding metallic stent, BTS: Bridge to surgery, LBO: Large bowel obstruction, RCT: Randomised controlled trial, CRC: Colorectal cancer, OS: Overall survival, DFS: Disease-free survival, QoL: Quality of life

Authors & Year	Study Design	Population (n, criteria)	Intervention (SEMS as BTS)	Comparator (Emergency Surgery)	Primary Outcomes	Secondary Outcomes	Functional Outcomes (Surrogates)	Oncological Outcomes	Key Findings
van Hooft JE et al., 2011 [9]	RCT	n=98; acute left-sided malignant LBO	SEMS + elective surgery	Emergency resection	Mortality, morbidity, perforation	Stoma rate	Clinical decompression	Not primary endpoint	↑ perforation; no mortality benefit
Wang Y et al., 2019 [10]	Retrospective cohort	n=78; patients with acute obstructive colorectal cancer (left-sided predominance)	SEMS as BTS	Emergency surgical resection	Postoperative morbidity, mortality	Stoma rate, hospital stay	Bowel decompression, time to surgery	OS, DFS	↓ stoma rate, ↓ postoperative complications, shorter hospital stay with SEMS; no significant difference in long-term survival
Ho KS et al., 2011 [11]	RCT	n=47; obstructing CRC	Endoscopic SEMS + elective surgery	Emergency surgery	Morbidity	Stoma rate, hospital stay	Bowel decompression, time to surgery	Not reported	↓ stoma rate, ↓ hospital stay, similar morbidity and mortality with SEMS
Sloothaak DA et al., 2014 [12]	Cohort	n=73; malignant LBO	SEMS as BTS	Emergency surgery	Long-term survival	Recurrence	Not reported	OS, DFS	No oncological difference
Arezzo A et al., 2017 [13]	RCT	n=144; obstructive left-sided CRC	SEMS as BTS	Emergency surgery	Morbidity	Stoma rate, QoL	QoL improvement	OS, recurrence	↓ stoma rate; similar outcomes
Katsuki R et al., 2020 [14]	Cohort	n≈212; malignant LBO	SEMS as BTS	Emergency surgery	Mortality	Stoma rate hospital stay	Relief of obstruction, bowel decompression	Overall survival, recurrence	SEMS associated with lower stoma formation, shorter hospital stay, similar short-term morbidity and long-term oncological outcomes
Siddiqui A et al., 2017 [15]	Multicenter trial	n=167; adults with acute proximal malignant colonic obstruction, palliative intent	SEMS as BTS	Emergency surgery	Long-term survival	Stoma rate	Relief of obstruction, time to oral intake	Overall survival	SEMS associated with shorter hospital stay, fewer immediate complications, comparable long-term survival

Risk of Bias Assessment

Tables 2, 3 present the methodological quality of the included studies, evaluated using the RoB 2 tool for randomised controlled trials (RCTs) and ROBINS-I for observational studies. Among the RCTs, van Hooft et al. (2011) showed some concerns in randomisation and selective reporting due to early termination and limited sample size, while deviations from intended interventions, missing outcome data, and outcome measurement were low risk [9]. Ho KS et al. (2011) demonstrated some concerns in randomisation and a higher risk of bias in reported results, likely related to small sample size and limited reporting [11]. In contrast, Arezzo et al. (2017) were low risk across all domains, reflecting their robust multicentre design [13]. For observational studies, Sloothaak et al. (2014) had a moderate overall risk, primarily from residual confounding in post-RCT follow-up [12]. Katsuki R et al. (2020) carried a serious risk due to the confounding inherent in its non-randomised design, with moderate concerns in outcome assessment [14]. Wang Y et al. (2019) were at moderate risk, influenced by potential confounding, selection bias, and limitations of retrospective data collection [10]. Finally, Siddiqui A et al. (2017) had a moderate risk, mainly from potential attrition and long-term data completeness issues [15].

**Table 2:** Risk of bias assessment of randomised controlled trials using Cochrane RoB 2 tool RoB 2: Risk of Bias 2 tool.

Study	Randomisation Process	Deviations from intenstion	Missing Outcome Data	Measurement of Outcomes	Reported Results selection	Overall Risk of Bias	Justification
van Hooft JE et al., 2011 [9]	Some concerns	Low	Low	Low	Some concerns	Some concerns	Early termination; limited sample size
Ho KS et al., 2011 [11]	Some concerns	Low	Low	Low	Some concerns	Some concerns	Small sample size and limited reporting detail; otherwise prospective randomized design with acceptable methodology
Arezzo A et al., 2017 [13]	Low	Low	Low	Low	Low	Low risk	Robust multicenter design

**Table 3:** Risk of bias assessment of observational studies using ROBINS-I tool ROBINS-I: Risk Of Bias In Non-randomised Studies of Interventions.

Study	Confounding	Selection Bias	Classification of Intervention	Deviations from Interventions	Missing Data	Measurement of Outcomes	Reported Results selection	Overall Risk of Bias	Justification
Wang Y et al., 2019 [10]	Moderate	Moderate	Low	Low	Low	Moderate	Low	Moderate	Retrospective design with potential confounding and selection bias; outcome assessment partly dependent on clinical records
Sloothaak DA et al., 2014 [12]	Moderate	Low	Low	Low	Low	Low	Low	Moderate	Post-RCT follow-up; residual confounding
Katsuki R et al., 2020 [14]	Serious	Moderate	Low	Low	Low	Moderate	Low	Serious	Non-Retrospective design with potential residual confounding despite comprehensive data collection
Siddiqui A et al., 2017 [15]	Moderate	Low	Low	Low	Low	Low	Low	Moderate	Multicenter observational design; possible differences in baseline comorbidities and tumor burden

Discussion 

Acute malignant obstruction of the left colon is a high-risk surgical scenario, carrying significant morbidity and mortality due to progressive colonic distension, perforation risk, and metabolic derangements [1,2]. Traditional management with emergency resection is often associated with considerable clinical burden, including high rates of stoma formation, postoperative complications, and prolonged hospitalisation, particularly among elderly or comorbid patients [3]. These challenges highlight the need for alternative strategies that can mitigate perioperative risk and improve patient outcomes.

SEMS have emerged as an effective alternative bridge-to-surgery option, allowing decompression of the obstructed colon, correction of fluid and electrolyte disturbances, and stabilisation of the patient prior to elective surgery [4]. By converting an urgent operation into a planned procedure, SEMS facilitates optimisation of perioperative care and may reduce immediate postoperative complications. Randomised studies support these benefits: Ho KS et al. (2011) reported that SEMS reduced stoma formation and allowed effective bowel decompression, with morbidity similar to emergency surgery [11]. Arezzo et al. (2017) demonstrated comparable findings in a multicentre RCT, showing lower stoma rates and improved quality-of-life outcomes, without negatively affecting oncological endpoints [13]. Observational data, such as the study by Katsuki R et al. (2020), also indicate lower stoma formation and shorter hospital stays with SEMS, with comparable short-term morbidity and long-term survival [14]. Evidence on short-term morbidity and mortality is mixed. van Hooft et al. (2011) observed increased perforation risk with SEMS, though mortality did not differ significantly [9]. Wang Y et al. (2019) did not show clear benefits in morbidity or mortality, likely influenced by limited sample size and early study termination [10]. Overall, SEMS appears safe when performed by experienced operators, with the main advantage being improved perioperative management and reduced stoma requirement rather than a direct survival benefit.

Long-term oncological outcomes are generally similar between SEMS and emergency surgery. Studies by Sloothaak et al. (2014) and Siddiqui et al. (2017) found no significant differences in overall survival, disease-free survival, or recurrence rates [12,15]. Arezzo et al. (2017) also reported no detrimental effects on oncological outcomes, alleviating concerns about potential tumour spread or delays in definitive surgery [13]. Nevertheless, the current literature has limitations. There is considerable heterogeneity in study design, patient selection, stent technique, and outcome reporting, restricting robust quantitative comparisons. Many RCTs are small or prematurely terminated [9-11], while observational studies remain vulnerable to confounding and selection bias [12,14,15]. Reporting of functional outcomes, quality-of-life measures, and long-term follow-up is inconsistent. Future research should focus on well-powered multicentre RCTs with standardised outcomes, including functional recovery, patient-reported quality of life, and cost-effectiveness.

In summary, SEMS as a bridge to surgery offers meaningful advantages, particularly in lowering stoma rates, optimising perioperative care, and facilitating elective resection, without compromising long-term oncological outcomes. In appropriately selected patients and when performed by experienced clinicians, SEMS represents a safe and effective alternative to emergency surgery for left-sided malignant colonic obstruction.

## Conclusions

Colonic stenting as a bridge to surgery is a safe and effective strategy for managing acute left-sided malignant large bowel obstruction, offering clear short-term benefits compared with emergency resection. SEMS significantly reduces stoma formation and facilitates elective surgical planning, allowing optimisation of the patient’s clinical condition prior to definitive resection. Short-term morbidity and mortality are comparable between SEMS and emergency surgery, while long-term oncological outcomes, including overall survival, disease-free survival, and recurrence, remain similar across both methods. Although SEMS carries risks such as perforation or stent-related complications, careful patient selection and procedural expertise can mitigate these adverse events. Overall, SEMS as a bridge to surgery provides a valuable alternative to emergency resection, aligning with the dual goals of improving perioperative outcomes and maintaining oncological safety in patients with left-sided malignant large bowel obstruction.
